# Cognitive and psychiatric signs revealing Sneddon syndrome: A case report

**DOI:** 10.1002/ccr3.8013

**Published:** 2023-10-04

**Authors:** Mehdi Karoui, Emna Baklouti, Dina Ben Mohamed, Hend Riahi, Mouna Chelli‐Bouaziz

**Affiliations:** ^1^ Razi Hospital Psychiatry G oranges street Manouba Tunisia; ^2^ Neurology La Rabta Tunisia Tunis Institut National de Neurologie Mongi‐Ben Hamida Tunis Tunisia; ^3^ Kassab Institute Ariana Tunisia; ^4^ Mohammed Kassab National Institute of Orthopaedics Tunis Tunisia

**Keywords:** central nervous system vasculitis, livedo, psychosis, Sneddon syndrome

## Abstract

**Key Clinical Message:**

The diagnosis of Sneddon Syndrome should be considered in adults with young‐onset dementia accompanied by neuropsychiatric signs and livedo racemosa. Magnetic resonance imaging and cerebral angiography are essential. A cutaneous biopsy may help in the diagnosis.

**Abstract:**

Sneddon syndrome (SS) is a clinical entity corresponding to a noninflammatory thrombotic vasculopathy that typically includes livedo racemosa and cerebrovascular ischemia. Psychiatric symptoms with cognitive impairment often occur but are rarely the inaugural symptoms. We present a case of secondary SS in a 45‐year‐old man in whom dementia and psychosis revealed the disease.

## Graphical abstract

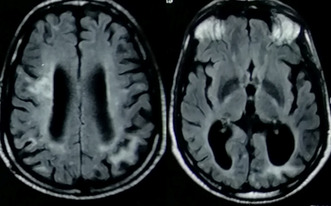

## 1 INTRODUCTION

SS is a rare noninflammatory thrombotic vasculopathy affecting the small and medium arteries of the skin and brain.^1^ Its Orpha number is ORPHA820.^2^ In almost half of all cases, antiphospholipid syndrome is detected.^2^, ^3^ This report aimed to describe a case of SS with cognitive impairments and psychotic symptoms as the inaugural symptoms.

## 2 CASE REPORT

A 45‐year‐old Tunisian man presented with a three‐year history of memory impairment, speech disorder, confusion, and hallucinations. The family also described a loss of autonomy for routine tasks, disordered sleep, and labile mood. The initial diagnosis was a major depressive episode with psychotic features, according to the diagnostic and statistical manual of mental disorders: DSM‐5 criteria.^4^ The patient received fluoxetine (20 mg/day) and olanzapine (10–20 mg/day), but there was no improvement. While examining the patient, we discovered a livedo racemosa appearance located mainly on the trunk and upper extremities. The patient also described Raynaud's phenomenon in his fingers. Neurological and psychiatric examinations found delusions of persecution with auditory and visual hallucinations, mild dysarthria, and pyramidal syndrome. The patient scored 19 points out of 30 on the Mini‐Mental Status Examination with dysexecutive syndrome and hippocampal amnesic syndrome. The family history revealed stroke and hypertension. The immunological workup revealed positive antiphospholipid antibodies.

**FIGURE 1:**
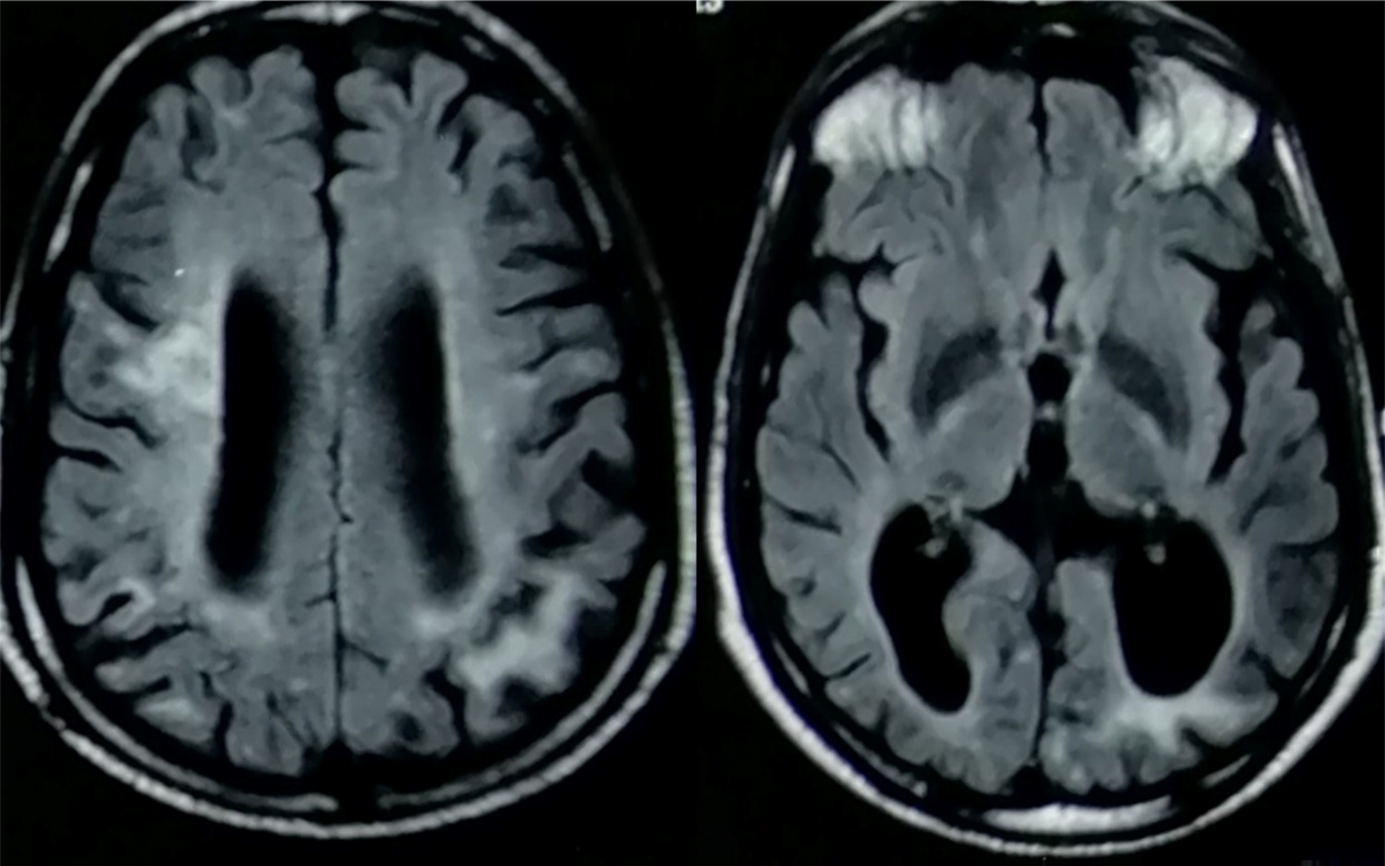
Brain MRI showed multiple ischemic lesions on T2‐weighted, some Gadolinium‐enhanced cortical–subcortical regions and on the left occipital lobe and left and right parietal lobes, and diffuse brain atrophy.

Brain MRI [Figure 1] showed multiple ischemic lesions on T2‐weighted imaging, some gadolinium‐enhanced cortical–subcortical regions, and diffuse atrophy of the left occipital lobe and left and right parietal lobes.

**FIGURE 2:**
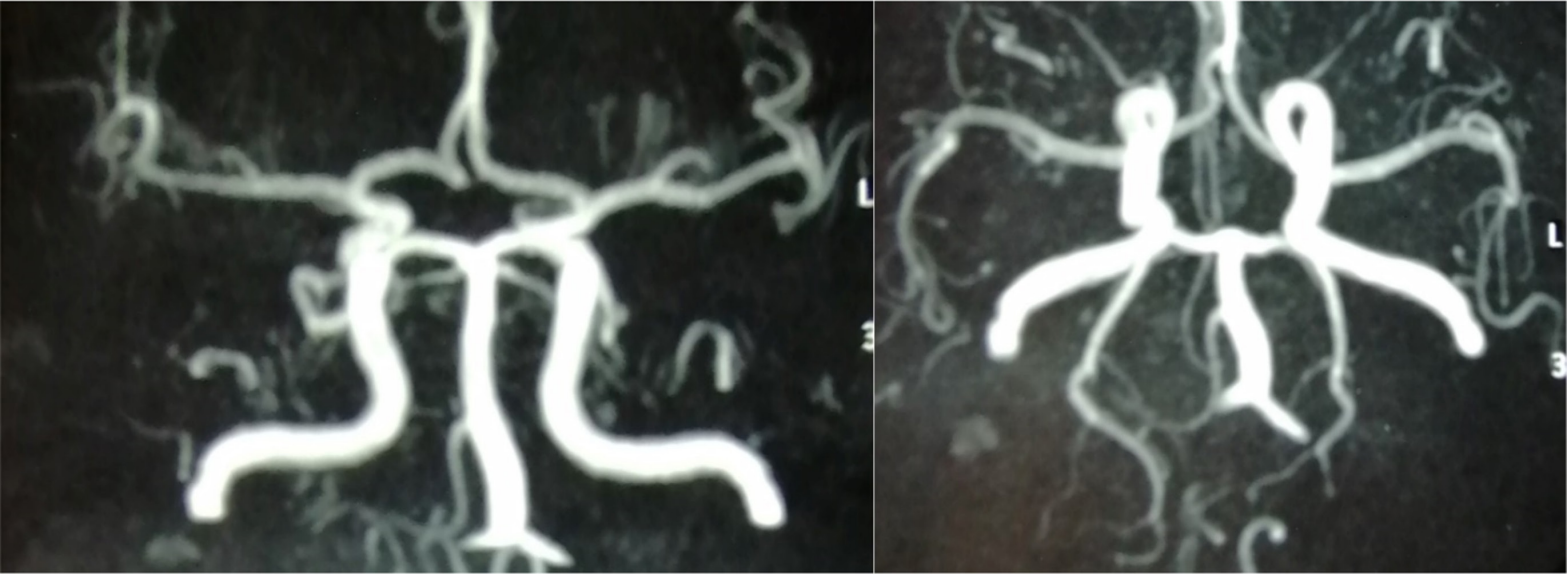
Angiography shows rarefaction of the distal branches of the middle cerebral artery.

Cerebral angiography [Figure 2] revealed decreased vessel diameter and parietal irregularities in the distal cerebral arteries, suggestive of vasculitis.

The patient was discharged on acetylsalicylic acid (100 mg/day), oral corticosteroids (prednisolone 60 mg/d), and oral anticoagulants (acenocoumarol 4 mg/day) with a diagnosis of SS. Clinical stability was recorded during 12 months of follow‐up. In particular, there was no recurrence of stroke. No deterioration in autonomy or cognitive functions was observed.

## 3 DISCUSSION

The analysis of the data and the presentation of the results were performed according to the Declaration of Helsinki statement of ethical principles.

SS is a rare entity, with an annual incidence of four cases per million.^5^ It is a clinical diagnosis defined by the association of livedo racemosa with brain strokes.^2^ This entity has been associated with several etiologies.^2^ Schellong et al.^6^ have made a distinction between a primary and a secondary type of SS. The latter is related to systemic lupus erythematosus or antiphospholipid syndrome.^2^ A thrombophilic form has also been described by the same authors.^6^

Clinically, dementia is rarely inaugural in SS. It may simulate the Alzheimer's type of dementia, making the diagnosis more difficult.^3^ Concentration, attention, memory, visual perception, and visuospatial construction are the most commonly described cognitive impairments.^3^ The pathophysiology of the neuropsychiatric symptoms is based on repeated unrecognized cerebral ischemic accidents that cause a deterioration in intellectual functions.^3^

The course of dementia in SS is progressive. It is preceded in half of all cases by a transient cerebral accident.^5^ Cognitive disorders are another hallmark of the disease, which justifies close neurological monitoring in any patient presenting with an isolated case of livedo racemosa.^3^

Cases of other psychiatric symptoms in patients who were diagnosed with SS have been reported in the literature. We found two case reports regarding suicide attempts in patients with SS. In the first case, it was a suicide attempt in a patient with bipolar II disorder for whom oral olanzapine treatment was not effective; this episode ultimately led to a diagnosis of SS.^7^ The second case report discussed the case of a woman with SS who made a suicide attempt in the context of an episode of psychosis.^8^ She was ultimately put on a low dose of chlorpromazine and showed a good response. The authors hypothesized that the psychiatric symptoms were secondary to SS. In our case, the psychiatric symptoms included delusions of persecution with auditory and verbal hallucinations.

Psycho‐cognitive signs generally occur after years, but cases of dementia and mood disorder secondary to subclinical recurrent strokes have been reported without preceding episodes of a focal neurological deficit.^9^ In one case report, cognitive impairment was the first clinical presentation of SS. The authors of the case report explained it as vascular dementia, believing it could be secondary to silent strokes that went unnoticed.^2^, ^9^

Radiologically, cerebral angiography is the gold standard for diagnosing SS.^9^ Angiography demonstrates better resolution for small‐ to medium‐sized vessels than computed tomography (CT) or MRI. In the early stages, angiopathy affects only the capillaries and arterioles, so scans may appear normal.^5^ They may remain normal in up to half of all cases. The other half shows obstruction that is sometimes responsible for a collateral arteriolar network.^9^ SS can also be detected with MRI or CT scans, with MRI showing better sensitivity than CT scans.^9^ MRI may show lacunar infarcts and signal alterations in the periventricular white matter, suggestive of chronic ischemia and cortical atrophy.^10^ Three aspects of cerebral infarction have been described.^10^ In the case of occlusion of a middle‐sized artery, a sizable cortical–subcortical infarction is observed. If a superficial perforating artery is involved, a smaller distal infarct is seen. More rarely, if a deep perforating artery is thrombosed, a deep white matter infarct is observed.^9^, ^11^ In addition, strokes may be supra‐ and infra‐tentorial, but the basal ganglia and cerebellum are rarely involved.^12^

In terms of histology, a cutaneous biopsy may show occlusion of the arterioles with intimal proliferation.^13^ For several authors, these histological findings have been of little help in making the diagnosis of SS because they are neither specific nor sensitive.^14^, ^15^ No cutaneous biopsy was performed in our case.

## 4 CONCLUSION

When encountering an adult with young‐onset cognitive dysfunction or psychiatric symptoms and dermatological abnormalities, the diagnosis of SS should not be overlooked. If strokes are found, especially in light of the unusual livedo racemosa skin rash, further workup should be performed to detect this autoimmune disease. MRI and cerebral angiography are essential. A cutaneous biopsy may help in the diagnosis.

## AUTHOR CONTRIBUTIONS

**Mehdi Karoui:** Conceptualization; investigation; project administration; validation; writing – review and editing. **Emna Baklouti:** Writing – original draft; writing – review and editing. **Dina Ben Mohamed:** Writing – original draft. **Hend Riahi:** Resources; supervision; validation; writing – original draft. **Mouna Chelli‐Bouaziz:** Software; validation; visualization.

## FUNDING INFORMATION

None.

## CONFLICT OF INTEREST STATEMENT

The authors report no conflicts of interest.

## CONSENT

Written informed consent was obtained from the patient to publish this report in accordance with the journal's patient consent policy.

## Data Availability

Data sharing is not applicable to this article, and data used to support this study are included within the article.
